# Metahemoglobinemia secundaria al uso de primaquina en un caso pediátrico de malaria

**DOI:** 10.7705/biomedica.7194

**Published:** 2025-03-28

**Authors:** Sara Puerta, Hardenson Rodríguez

**Affiliations:** 1 Especialización en Pediatría, Universidad Pontificia Bolivariana, Medellín, Colombia Universidad Pontificia Bolivariana Universidad Pontificia Bolivariana Medellín Colombia; 2 Servicio de Pediatría, Hospital Pablo Tobón Uribe, Medellín, Colombia Hospital Pablo Tobón Uribe Hospital Pablo Tobón Uribe Medellín Colombia; 3 Especialización en Oncohematología Pediátrica, Universidad Nacional de Colombia, Bogotá, D. C., Colombia Universidad Nacional de Colombia Universidad Nacional de Colombia Bogotá, D. C. Colombia

**Keywords:** metahemoglobina, metahemoglobinemia, cianosis, malaria, primaquina, pediatría, Methemoglobin, methemoglobinemia, cianosis, malaria, primaquine, pediatrics

## Abstract

La metahemoglobinemia es el resultado del aumento de la concentración de metahemoglobina en la sangre, lo cual impide una adecuada liberación del oxígeno en los tejidos. Se considera una condición poco frecuente que requiere de una gran sospecha diagnóstica.

Se reporta el caso de un paciente con malaria que presentó cianosis e hipoxemia como manifestación de metahemoglobinemia· esta última causada por el consumo de primaquina. El paciente mejoró tras la suspensión del antimalárico y la administración de ácido ascórbico.

## Abstract (en)

Methemoglobinemia results from an increased concentration of methemoglobin in the blood· impairing oxygen delivery to tissues. It is considered a rare condition that requires a high diagnostic suspicion.

We report the case of a pediatric patient with malaria who presented with cyanosis and hypoxemia as manifestations of methemoglobinemia induced by primaquine treatment. The patient responded adequately after antimalarial drug suspension and ascorbic acid administration.

La metahemoglobinemia es una entidad clínica que se produce debido a grandes concentraciones de metahemoglobina en la sangre· lo cual impide una adecuada liberación de oxígeno en los tejidos ^1^. Es una condición poco frecuente que requiere una gran sospecha diagnóstica. Tiene dos formas de presentación: adquiridas y congénitas; las adquiridas son las más frecuentes· especialmente aquellas secundarias a la exposición a sustancias que favorecen la oxidación de la hemoglobina ^2^. Las manifestaciones clínicas son muy variables y dependen de la causa; su espectro clínico abarca desde pacientes asintomáticos hasta aquellos con compromiso sistémico grave ^3^.

Actualmente, las guías para la atención integral de los pacientes con malaria mencionan la metahemoglobinemia como uno de los potenciales eventos adversos de la primaquina. Sin embargo, no establecen directrices definidas para su tratamiento.

Dada la relevancia y el estado actual del conocimiento sobre el tema, se presenta el caso de un niño en edad preescolar que desarrolló metahemoglobinemia secundaria al uso de antimaláricos. Se describen las características clínicas y el abordaje diagnóstico realizado.

## Presentación del caso

Se trata de un paciente de sexo masculino, de 3 años y 9 meses de edad, residente en Frontino (Antioquia), municipio considerado como zona endémica para malaria. No se mencionaron antecedentes patológicos ni familiares de importancia para el caso actual.

El paciente fue llevado a consulta al hospital local por un cuadro clínico de 12 días de evolución de picos febriles cuantificados, asociados con astenia, mialgias y adinamia.

En la primera evaluación, se encontró evidencia de hepatomegalia. Los exámenes de laboratorio practicados incluyeron un hemograma que mostró hemoglobina de 10,7 mg/dl, hematocrito del 34 % y pancitopenia (4.000 leucocitos/μl, 2.300 neutrófilos/μl, 1.600 linfocitos/μl y 29.000 plaquetas/μl), sin elevación de las aminotransferasas ni patrón colestásico (aspartato aminotransferasa de 19,1 U/L, alanina aminotransferasa de 29,6 U/L, bilirrubina total de 0,65 mg/dl y bilirrubina directa de 0,37 mg/dl).

La gota gruesa fue positiva para *Plasmodium vivax* con parasitemia de 801 parásitos/μl. Después del diagnóstico y tras 12 días de síntomas, el paciente fue tratado con 150 mg diarios de cloroquina (10 mg/kg) y 10 mg de primaquina por día. Esta última correspondió a más del doble de la dosis recomendada para su edad y peso, pues recibió 0,6 mg/kg/día, cuando la dosis correcta -según las guías de práctica clínica- es de 0,25 mg/kg/día. El mismo día del diagnóstico e inicio del tratamiento, el paciente fue trasladado a un hospital de mayor complejidad, con diagnóstico de malaria no complicada, pero con signos de alarma.

El niño fue hospitalizado en condiciones generales aceptables, con la hepatomegalia ya descrita y sin hallarse otras alteraciones en el examen físico. En los exámenes de laboratorio de ingreso se reportó persistencia de la anemia (hemoglobina de 9,9 mg/dl y hematocrito de 29,6 %) y trombocitopenia (31.000 plaquetas/μl) y leucocitos normales (6.400 células/μl). En los gases arteriales, se informó: pH de 7,44, presión parcial de CO_2_ de 33 mm Hg y de O_2_ de 76 mm Hg, HCO_3_ de 22,4 mEq/L y base exceso de -1,3 mmol/L.

El examen de gota gruesa resultó nuevamente positivo para *P. vivax* (7.200 parásitos/μl). Se continuó el tratamiento con 150 mg diarios de cloroquina (10 mg/kg) en el segundo día y 75 mg diarios (5 mg/kg) en el tercer día, hasta completar tres dosis. Se ajustó la dosis de primaquina a 5 mg por día (0,33 mg/kg) para continuar a partir del día siguiente, hasta completar 14 días.

El paciente finalizó el tratamiento con cloroquina y continuó con primaquina. Sin embargo, al cuarto día de tratamiento, después de haber recibido cuatro dosis de primaquina, presentó hipoxemia de hasta el 76 %, con ligera cianosis peribucal no asociada con signos de dificultad respiratoria, sin hallazgos patológicos al momento de la auscultación pulmonar. Se inició oxígeno suplementario con poca mejoría, manteniéndose saturaciones del 85 % con máscara sin reinhalación.

La radiografía de tórax mostró hallazgos normales y en otra prueba de gases arteriales se encontró: pH de 7,5, presión parcial de CO_2_ de 30 mm Hg y de O_2_ de 129 mm Hg, HCO_3_ de 25,5 mEq/L, base exceso de 0,6 mmol/L, lactato de 0,9 mmol/L y hemoglobina de 9,5 mg/dl, que correspondieron a alcalosis respiratoria con lactato normal, hemoglobina estable y presión parcial de oxígeno estándar. Sin embargo, llamaba la atención la diferencia en la saturación de oxígeno obtenida entre los gases arteriales y la pulsoximetría.

Por esta razón, ante la sospecha de metahemoglobinemia, se solicitó cuantificar la metahemoglobina en la prueba de control de gases arteriales a las seis horas. Como resultado, se reportó un valor de metahemoglobina del 17,1 % y aumento de la presión parcial de oxígeno (pH de 7,48, presión parcial de CO_2_ de 28 mm Hg y O_2_ de 301 mm Hg, HCO_3_ de 23,2 mEq/L). Con estos hallazgos, se diagnosticó metahemoglobinemia secundaria al uso de antimaláricos y se suspendió la administración de primaquina.

El paciente fue valorado por infectología pediátrica y, con el nuevo diagnóstico y la buena respuesta a la terapia antimalárica (examen de gota gruesa negativa para *P. vivax* al tercer día de tratamiento), se decidió no completar el tratamiento con primaquina y se indicó continuar con el seguimiento clínico haciendo una prueba de gota gruesa cada dos semanas durante un mes.

Por la persistencia de la anemia documentada en los exámenes previos de laboratorio y a pesar de no tener evidencia clínica de hemólisis, durante la evolución se descartó una deficiencia enzimática de glucosa-6-fosfato deshidrogenasa mediante la cuantificación de la enzima por espectrofotometría cinética. Como resultado, se obtuvo un valor de 18,8 U/gHb, dentro del rango de normalidad (valores de referencia entre 6,9 y 20,5 U/gHb).

El paciente continuó hospitalizado en la institución. Al noveno día, se cuantificó la concentración de metahemoglobina en la prueba de control de gases arteriales, en la que se observó disminución de estos: pH de 7,45, presión parcial de CO_2_ de 30 mm Hg y O_2_ de 292 mm Hg, HCO_3_ de 21,1 mEq/L y metahemoglobina del 8,4 %. A pesar de ello, y aún con uso de oxígeno suplementario con máscara sin reinhalación, no se logró mejorar la saturación de oxígeno.

Por esta razón, el Servicio de Toxicología indicó iniciar tratamiento con ácido ascórbico y planteó el uso de azul de metileno. No obstante, dada la mejoría clínica obtenida con el ácido ascórbico, no se administró el azul de metileno. Se logró suspender completamente el oxígeno suplementario a medida que mejoró su saturación. En los últimos exámenes de laboratorio, la prueba de gota gruesa resultó negativa para *Plasmodium spp*. Finalmente, el paciente fue dado de alta con control de gota gruesa seriado -como fue indicado por infectología- y administración de ácido ascórbico durante una semana.

## Discusión

La metahemoglobina es una forma alterada de la hemoglobina. Se produce como consecuencia de la oxidación del hierro del grupo hemo, que pasa de un estado ferroso (Fe^2+^) a uno férrico (Fe^3+^) ^2^. El proceso de oxidación lleva a la pérdida del electrón necesario para formar el enlace con el oxígeno, razón por la cual, la metahemoglobina es incapaz de transportarlo. Además, el grupo hemo férrico dentro de la hemoglobina tetramérica provoca que los tres grupos hemo ferrosos aumenten su afinidad por el oxígeno, lo cual incrementa la disociación de la hemoglobina. Esto afecta aún más la entrega de oxígeno a los tejidos y, como resultado, causa anemia funcional ^3^.

La concentración de metahemoglobina en sangre está regulada por la enzima citocromo b5 reductasa (Cyb5R) con la ayuda de la coenzima nicotinamida adenina dinucleótido reducida (NADH) y, en condiciones normales, no supera el 1 %. Sin embargo, puede aumentar debido a causas hereditarias o adquiridas.

Entre las causas hereditarias, se encuentran la deficiencia enzimática de Cyb5R, la enfermedad de la hemoglobina M y la deficiencia de citocromo B5 ^4^.

En la metahemoglobinemia hereditaria, la deficiencia de la Cyb5R causa la mayoría de los casos, ya que es una condición autosómica recesiva con dos variantes ^5^. La variante 1 se encuentra en el 90 % de los casos, produce una inestabilidad enzimática restringida a los glóbulos rojos, asociada con concentraciones de metahemoglobina entre el 10 y el 35 %. Es una condición clínica de buen pronóstico, con cianosis ocasional, pero, por lo demás, asintomática ^6^. La variante 2 se encuentra en el 10 % de los casos, en los cuales 8 % a 40 % de la hemoglobina está en forma de metahemoglobina. Se caracteriza por una baja actividad enzimática de las células no eritroides y causa síntomas neurológicos y retraso en el desarrollo, por lo cual acorta la esperanza de vida e, incluso, puede ser fatal en el primer año de vida en la mayoría de los casos ^1^.

La enfermedad de la hemoglobina M es una condición autosómica dominante, secundaria a la sustitución de una histidina por una tirosina, lo que genera variantes estructurales en las cadenas proteicas de las globinas alfa, beta o gamma, las cuales inducen la oxidación del hierro ^7^.

Finalmente, se encuentra la deficiencia del citocromo B5, sustrato de la enzima Cyb5R y causante de la forma más rara de metahemoglobinemia congénita ^3^. Estas condiciones clínicas no fueron evaluadas en el paciente.

La mayoría de los casos de metahemoglobinemia se deben a causas adquiridas, inducidos por sustancias exógenas. Pueden producirse en el contexto de un envenenamiento, el uso de medicamentos (cuadro 1) a dosis estándar o por sobredosis de estos ^8^.

**Cuadro 1:** Medicamentos que pueden causar metahemoglobinemia ^2^^,^^13^^,^^20^

Medicamentos	
Ácido nalidíxico	Metoclopramida
Anestésicos locales tópicos (benzocaína, lidocaína, prilocaína)	Nitratos, nitritos
Azul de metileno	Nitroglicerina
Bupivacaína	Nitroprusiato
Cafeína	Norfloxacina
Cefadroxilo	Óxido nítrico inhalado
Cefazolina	Primaquina
Cloroquina	Rasburicasa
Cianocobalamina	Rifampicina
Dapsona	Subsalicilato de bismuto
Dexametasona	Sulfadiazina
Difenhidramina	Sulfas (sulfametoxazol)
Fenilefrina	Sulfonamidas
Fenitoína	Tetraciclina
Hidroxicobalamina	Trimetroprim
Isoniazida	Valproato
Magnesio	Zinc
Metilprednisolona	

Entre las principales causas está el uso de dapsona, anestésicos tópicos (benzocaína o lidocaína), óxido nítrico inhalado, drogas de abuso que contienen nitritos como el nitrito de amilo (cada vez más usado en adolescentes y adultos jóvenes), tintes de anilina presentes en pesticidas o algunas sustancias químicas y antimaláricos como la primaquina. Esta última fue la causante de la condición descrita, en la que se observó la relación causal entre la administración del medicamento y las manifestaciones clínicas del paciente ^9^^,^^10^.

En la población pediátrica, la metahemoglobinemia debe sospecharse en pacientes que presenten cianosis o hipoxemia inexplicables, en ausencia de enfermedades respiratorias o cardiacas y sin mejoría con el oxígeno suplementario ^11^. Esto sucedió en el caso expuesto, luego de descartar primero otras causas, como el potencial compromiso pulmonar por la malaria. Generalmente, en las formas adquiridas de metahemoglobinemia, la cianosis suele ser reciente, de inicio súbito y transitoria, posterior a la exposición del paciente a una sustancia oxidante.

En las formas hereditarias, lo habitual es que se presenten desde el nacimiento y persistan a lo largo de la vida. Incluso, dependiendo del tipo de deficiencia, podrían acompañarse de otros síntomas como alteraciones neurológicas, del desarrollo y retraso en el crecimiento ^12^. Se requiere una gran sospecha clínica y una evaluación oportuna, ya que es una condición potencialmente mortal. La gravedad de los síntomas se correlaciona con la concentración de metahemoglobina en sangre; generalmente, los individuos afectados manifiestan mayor sintomatología con niveles superiores al 30 %, mientras que valores mayores del 70 % son letales ^2^.

En el paciente descrito, con el diagnóstico oportuno se logró identificar el evento adverso, aun con valores bajos de metahemoglobina, pues la única manifestación clínica fue una cianosis sutil e hipoxemia.

Se requiere de un adecuado abordaje diagnóstico en esta condición. Para ello, es indispensable el examen de los gases arteriales con la cuantificación de la concentración de metahemoglobina. En esta prueba, los hallazgos más frecuentes son la acidosis metabólica, secundaria a una inadecuada entrega de oxígeno a los tejidos, lo que favorece el metabolismo anaerobio, y una mayor tasa de disociación de la hemoglobina, además de la diferencia en la saturación de oxigenación por la disminución de su transporte ^13^.

El tratamiento inicial se basa, principalmente, en medidas de soporte y en la eliminación de la exposición al agente oxidante. El azul de metileno, o cloruro de metiltioninio, un conocido colorante orgánico, participa como cofactor de la enzima NADPH-metahemoglobina reductasa. Actúa aceptando un electrón de la NADPH para convertirse en su forma activa que es el azul de leucometileno, capaz de reducir el hierro del grupo hemo -del estado férrico (Fe^3+^) al estado ferroso (Fe^2+^)-, lo cual disminuye la concentración de metahemoglobina ^10^.

El azul de metileno está indicado en la metahemoglobinemia sintomática con concentraciones de metahemoglobina superiores al 20 %, y en condiciones preexistentes con metahemoglobina superior al 10 %, como anemia y enfermedades cardíacas o pulmonares ^11^. Se recomiendan dosis de 1 a 2 mg/kg de una solución con 1 % de cloruro de metiltioninio, durante 3 a 5 minutos. Puede administrarse una segunda dosis de 1 mg/kg si persiste la cianosis después de la primera hora. La cantidad total de azul de metileno administrada no debe exceder los 7 mg/kg, ya que la bioacumulación del fármaco se ha relacionado con la inducción de metahemoglobinemia y hemólisis, porque produce una inversión de la acción reductora. El efecto clínico se espera entre los 10 y los 60 minutos después de la administración de la solución, con resolución de la cianosis. Se encuentra contraindicado en aquellos pacientes con deficiencia de glucosa-6-fosfato deshidrogenasa ^10^.

La terapia con azul de metileno no fue necesaria en el paciente, dada la evolución de la enfermedad y su buena respuesta a la terapia alternativa con el ácido ascórbico. Esta última es una vitamina hidrosoluble antioxidante, capaz de reducir el estrés oxidativo, actuando como un agente reductor no enzimático o utilizando el glutatión reducido como donante de electrones para eliminar los radicales libres y, así, disminuir la concentración de metahemoglobina. Además, se cree que reduce la metahemoglobina al estado ferroso (Fe^2+^), permitiendo nuevamente que el hierro se una y libere oxígeno ^14^.

El ácido ascórbico causa el fenómeno de hormesis que, al usarse en grandes concentraciones, puede inducir la producción de radicales libres y peróxido de hidrógeno (relacionados con la peroxidación lipídica y la oxidación de la hemoglobina), lo cual, a su vez, puede generar hemólisis intravascular. Por esta razón, se debe tener precaución con las dosis altas de vitamina C para evitar la hemólisis como complicación secundaria de estas.

La velocidad de reacción del ácido ascórbico suele ser más lenta y requiere de múltiples dosis para lograr reducir la concentración de metahemoglobina. Se usa como tratamiento adyuvante o como sustituto del azul de metileno cuando este está contraindicado ^15^. No hay una recomendación estándar en la literatura respecto a su dosificación en la población pediátrica, pero se aconsejan dosis diarias entre 25 y 50 mg/ kg, cada ocho horas durante una semana ^16^. Sin embargo, existe gran variabilidad en la dosificación reportada en la literatura, lo que sugiere que se necesita más investigación en este campo para determinar el esquema óptimo del tratamiento con ácido ascórbico en la metahemoglobinemia. Aun así, se han descrito resultados favorables con muy pocos efectos adversos. En el presente caso, el ácido ascórbico fue el único medicamento administrado al paciente, quien lo toleró bien, lográndose reducir los niveles de metahemoglobina y la resolución del cuadro clínico. No obstante, a la luz de la evidencia, la recomendación actual es usar ambas estrategias terapéuticas con el fin de potenciar los diferentes mecanismos de acción ^2^.

El paciente con malaria aquí descrito presentó hipoxemia y cianosis secundarias a la administración de primaquina. La escasa mejoría obtenida con el oxígeno suplementario y la diferencia en la saturación de oxígeno observada entre el examen de gases arteriales y el pulsoxímetro, permitieron sospechar el diagnóstico. Sus concentraciones de metahemoglobina fueron menores del 30 %. Se suspendió la primaquina por un descenso importante de la saturación de oxígeno y se consideró iniciar tratamiento con ácido ascórbico. No se utilizó azul de metileno dada la buena reacción ante la terapia establecida. Algunos de los casos descritos en la literatura presentaron complicaciones como insuficiencia renal aguda, hemólisis y otras condiciones clínicas, que no se desarrollaron en el presente caso.

Es esencial enfatizar en la importancia de la dosificación adecuada de los medicamentos, en particular de la primaquina, ya que, como se mencionó anteriormente y se evidenció en el caso clínico, las sobredosis de medicamentos son una de las principales causas de metahemoglobinemia. No obstante, no se pudieron descartar causas primarias relacionadas con alteraciones genéticas en este paciente, ni una anemia preexistente. Es importante tener en cuenta que la primaquina puede provocar una disminución transitoria de la concentración de hemoglobina, incluso con la dosis estándar. Por lo tanto, en pacientes con malaria complicada o con signos de alerta, es recomendable administrarla después de su recuperación.

Actualmente, no se tienen reportes de la incidencia ni de la prevalencia de la metahemoglobinemia como entidad clínica, debido a su baja frecuencia. De hecho, se cuenta con poca información sobre la metahemoglobinemia secundaria a la primaquina en la población pediátrica. Entre los casos reportados, se resalta el de un menor en edad preescolar con ingestión accidental de primaquina ^17^, quien presentó hipoxemia sin mejoría con el oxígeno suplementario. Inicialmente, fue tratado con azul de metileno, pero desarrolló hemólisis secundaria debido a una deficiencia de la enzima glucosa-6-fosfato deshidrogenasa. Por lo tanto, se continuó el tratamiento con ácido ascórbico, con el cual se obtuvo un descenso adecuado de la concentración de metahemoglobina y una buena evolución. Otros pacientes de edad adulta presentaron manifestaciones clínicas similares a las del presente caso, como hipoxemia y cianosis ^18^^-^^20^. En estos casos, se prefirió el uso de ácido ascórbico junto con la suspensión de la primaquina.

La cianosis y la hipoxemia son manifestaciones clínicas frecuentes en el paciente pediátrico; sus principales causas son las enfermedades respiratorias, entre las cuales predominan las infecciones. Estos signos también se pueden presentar en los pacientes con formas graves de malaria. Entre los diagnósticos diferenciales que se deben considerar en un paciente clínicamente estable, se encuentra la metahemoglobinemia, la cual se confirma con un examen de gases arteriales. El tratamiento depende de la causa; dado que la mayoría son secundarias, la suspensión del medicamento desencadenante es fundamental, además de la administración de azul de metileno, solo o en conjunto con ácido ascórbico. Una gran sospecha diagnóstica determinará el inicio oportuno de la terapia y el buen pronóstico de la enfermedad. En las zonas endémicas para malaria, se deben considerar los antimaláricos como potencial causa de la metahemoglobinemia.
